# Level of Control of Dyslipidemia Among Patients Followed in Family Medicine Clinics in Riyadh, Saudi Arabia

**DOI:** 10.7759/cureus.15504

**Published:** 2021-06-07

**Authors:** Amal Hadi, Mohammed A AlAteeq

**Affiliations:** 1 Family Medicine, Ministry of National Guard - Health Affairs, King Abdullah International Medical Research Center, Riyadh, SAU; 2 Family Medicine, Ministry of National Guard - Health Affairs, King Abdullah International Medical Research Center, King Saud Bin Abdulaziz University for Health Sciences, Riyadh, SAU

**Keywords:** hyperlipidemia, lipid profile, ascvd, atherosclerosis, cvd prevention

## Abstract

Background

Dyslipidemia is a well-established primary risk factor leading to atherosclerotic cardiovascular disease (ASCVD). Treatment with lifestyle modification and lipid-lowering agents has been shown to reduce ASCVD morbidity and mortality.

Objectives

To explore the level of dyslipidemia control among patients followed in family medicine (FM) clinics and describe the prescribing pattern of lipid-lowering agents.

Materials and methods

This is a chart review cross-sectional observational study conducted over 382 patients who were followed in FM clinics at King Abdulaziz Medical City for National Guard, Riyadh, Saudi Arabia, from January 2016 to January 2019. The data were extracted from the electronic medical record system (*BESTCare*) and analyzed using Statistical Package for the Social Sciences (SPSS), version 23 (IBM Corp., Armonk, NY) to look for the association.

Result

All patients had a reduction in their lipid parameters over the three years follow-up period. The mean low-density lipoprotein cholesterol (LDL-C) for the total sample was (2.783 ± 0.850) mmol/L. 82.1% were using a statin alone, 6% were using statin plus *fenofibrate*, and 12.8% were switched from one statin to another. Those who had statin plus *fenofibrate *and those switched from one statin to another had the most reduction in their LDL, TC, and TG.

Conclusion

Most of the patients visiting the Ministry of National Guard - Health Affairs (MNG-HA), Riyadh, Saudi Arabia, showed a continuous reduction in their lipid profile over the follow-up period; with better control for high-risk patients. Many factors may have contributed to the reduction, like the number of clinic visits, dietician, and health educator visits, along with the type of medication used.

## Introduction

Lipids play a major role in cell communication, hormone regulation, nutrient transportation, and many other essential roles within the human body [1]. Dyslipidemia, also known as the disorder of lipid metabolism, is a condition that results in high levels of total cholesterol (TC) and other lipids or fat particles, including triglycerides (TG), low-density lipoprotein cholesterol (LDL-C), and non-high-density lipoprotein cholesterol (non-HDL-C). The condition is also associated with lower levels of high-density lipoprotein cholesterol (HDL-C) [2]. This lipid imbalance and its high amount cause them to adhere to the wall of the arteries and restrict the blood flow, which in turn create a significant risk of primary and secondary cardiovascular events, including heart attack and stroke [3]. 

As many factors can affect the lipid profile, dyslipidemia is affected by multiple underlying secondary conditions such as diabetes mellitus, hyperthyroidism, and other liver dysfunction and inflammatory renal diseases [4]. These conditions act by impairing the body's functions and enhancing atherogenicity, which increases the burden of dyslipidemia and the risk of cardiovascular diseases [5]. Chronic hyperglycemia among diabetic patients enhances the atherogenicity of LDL-C. Obesity is another condition where dyslipidemia is a common comorbidity and significantly increases the risk of atherosclerosis and the incidence of coronary artery disease among this group [6]. Moreover, dyslipidemia is affected by secondary lifestyle risk factors such as smoking, alcohol consumption, physical inactivity, and poor diet [7].

As dyslipidemia is linked with many chronic non-communicable diseases, it is also associated with multiple burdens, such as higher substantial costs on health care systems and higher associated morbidities and mortality costs [8]. The condition can affect both genders and individuals at any age group; however, it is more common among the elderly and females than males due to the hormonal changes they experience throughout their life that alters their lipoprotein levels [9]. In Saudi Arabia, the lipid disorders prevalence was reported to be (40.0%), (17.0%), (13.8%), and (12.85%) for Low HDL-C level, hypertriglyceridemia, hypercholesterolemia, and hyper-LDL-C, respectively [10]. Another study indicated that coronary artery diseases might become a significant future health problem in Saudi Arabia as their results reported 54% Saudis with hypercholesterolemia and 40.3% with hypertriglyceridemia in urban and rural areas. The high prevalence of dyslipidemia predictors such as diabetes, smoking, hypertension (HTN), and physical inactivity contributes to the large percentage of Saudis with dyslipidemias, and their assessment and treatment needs prioritization to reduce the risk of associated morbidities and mortalities [11]. A large proportion of patients with dyslipidemia with high and very high atherosclerotic cardiovascular disease (ASCVD) risk in the Arabian Gulf are not at their recommended non-HDL-C therapeutic targets [12].

The Recommendations of the American College of Cardiology/American Heart Association to manage cholesterol levels among patients with ASCVD are dependent on the disease severity and the risk level and targets the reduction of (LDL-C) [7]. These recommendations are based on the measurements of lipids and lipoproteins and include a heart-healthy lifestyle, statin therapy, and non-statin therapy with moderate to high intensities. The application of statin therapy was proven effective among diabetic and non-diabetic patients in lowering LDL-cholesterol levels [13].

Persistence high cholesterol and lipids levels require action as they are life-threatening. The recent American Diabetic Association (ADA) guidelines emphasized that cardiovascular risk factors, including dyslipidemia, should be regularly evaluated in all patients with diabetes [14].

Special consideration and proper management should be taken for the elderly, women especially pregnant, and those with chronic inflammatory conditions [15]. Changing individual lifestyle into a healthy style is identified to be the primary contributor for lowering lipid levels, and was shown to reduce the incidence of coronary heart disease [16].

The aim of this study is to explore the level of control of dyslipidemia among patients following in family medicine (FM) clinics at National Guard health facilities in Riyadh, Saudi Arabia, and to describe the prescribing pattern of lipids lowering agents.

## Materials and methods

This is a chart review, cross-sectional and analytic-study conducted for patients followed at three large primary care centers at King Abdul-Aziz Medical City-National Guard (KAMC-NG) in Riyadh, Saudi Arabia. The three primary health care centers are: Health Care Specialty Center (HCSC), for all National Guard employee, military and civilian, serves a population of around 200,000; King Abdulaziz City Housing (Iskan), for officers and soldiers housing, serves a population of around 50,000; National Guard Comprehensive Specialized Clinic (NGCSC), for all National Guard employee military and civilian, which serves around 100,000. Each center provides primary curative and preventive health services and has a walk-in and booking appointment system for patient visits for acute and chronic medical conditions.

The study included adult patients of both genders diagnosed with dyslipidemia with a minimum of two visits to the clinic in the period from January 1, 2016, to January 1, 2019. Patients with the following comorbidities that affect lipid profile were excluded: hypothyroidism, familial hypercholesterolemia, nephrotic syndrome, obstructive liver disease, and post-renal transplantation patients. In addition, pregnant women, patients who cannot tolerate lipid-lowering agents such as patients of active liver disease, cirrhosis, or liver failure were excluded.

The sample size was calculated using the Openepi version 3 statistical package. The total population from the data provided by King Abdullah International Medical Research Center (KAIMRC) was 25,092, reported to have dyslipidemia from the three PHC centers. A sample size of 379 was estimated based on the population size (n = 25,092), with a 95% confidence interval and a 5% margin of error. The sample size was increased to 382 to compensate for possible missing data. Patients were selected from the list by simple random technique using a random number generator.

All patient’s data were retrieved from the electronic medical record system in National Guard health facilities (BESTCare). Data were collected using a data collection sheet, designed to meet the study objectives, and includes demographic data (age, gender, and body mass index (BMI), comorbidities, and the number of visits to the FM and dietitian clinic in the patient’s record), latest labs (lipid profile in mmol/L, LDL-C, HDL-C, TG, TC, TSH, liver enzymes: alanine transaminase (ALT) and aspartate transaminase (AST), hemoglobin A1C), current anti-lipid medications (type of the medication, date, and dose on initiation, and latest dose).

All statistical analyses were carried with Statistical Package for the Social Sciences (SPSS), version 23 (IBM Corp., Armonk, NY). Before the analysis, data were gathered and cleaned of any errors, and patients with significant missing data were ruled out. Continuous variables were described using means and standard deviation, while categorical variables were described using frequencies and percentages. Analytic statistics were carried out utilizing the T-test to determine if there is a difference between the means of the two groups. A p-value of ≤ 0.05 was regarded as statistically significant.

Ethical approval was obtained from the Institutional Review Board at King Abdullah International Medical Research Center. Data collection sheets were coded using three‑digit serial numbers and were maintained by the co‑investigator. Participants could not be traced after the collection of the datasheets. The study was carried out according to the principles of the Helsinki Declaration.

## Results

The total number of patients was 382. The mean age was 58.5 years ± 10.6 years, the youngest patient was 28 years, and the oldest was 91years, and the majority were females (62.8%, n = 240). On average, the patients were obese with a mean BMI of 32.3 kg/m^2^ ± 5.87 kg/m^2^. Almost half of the patients (51.3%, n = 196) had 10 or fewer follow-up visits to the FM clinic in their record. Most of the patients (95%, n = 363) had no visit to the dietician, and only (6.5%, n = 25) had visited the health educator. More than half of patients had diabetes (67.3%, n = 257), and (58.9%, n = 225) had HTN. In addition, only (2.6%, n = 10) had a stroke (Table 1). The study sample was further categorized into high-risk and low risk. The high-risk group represents patients with DM, HTN, and stroke, while the low-risk group represents patients with dyslipidemia only. The majority of patients were high-risk (81.4%, n = 311).

**Table 1:** Demographic data. HCSC: Health Care Specialty Center; NGCSC: National Guard Comprehensive Specialized Clinic; PHC: Primary Health Centre.

Characteristic	Frequency	%
Gender		
Females	240	62.8
Males	142	37.2
Clinic		
HCSC	238	62.3
Iskan	44	11.5
NGCSC	100	26.2
Clinical visits		
≥ 10 visits to PHC doctor	186	48.7
< 10 visits to PHC doctor	196	51.3
At least one visit to the dietician	363	95
At least one visit to the health educator	357	93.5
Comorbidities		
Diabetes mellitus	257	67.3
Hypertension	225	58.9
Stroke	10	2.6
Anti-lipid medication prescription		
Atorvastatin	289	75.7
Simvastatin	82	21.5
Rosuvastatin	15	3.9
Fenofibrate	24	6.3
No medication (diet and exercise only)	23	6
Anti-lipid medication trial and combination		
Only one statin drug inpatient record	310	81.2
Trial of more than one statin drug	49	12.8
On both statin and fenofibrate drugs	23	6
On fenofibrate only	1	0.3

The majority of patients (81.2%,) had only one statin drug in their record, 12.8% were switched from one statin to another, and 6% were on a diet and exercise only. The most used statin drug was atorvastatin (75.7%, n = 289), followed by simvastatin (21.5% n = 82), and the least used statin was rosuvastatin (3.9%, n = 15). Only (6%, n = 23) of patients were on both fenofibrate and statin drugs. While one patient (0.3%) was using fenofibrate drug only (Table 1).

Table 2 represents the mean of the latest lipid parameters for the total, high-risk, and low-risk groups. Overall, there was a reduction of lipid profile throughout the follow-up period, as shown in Table 3. Table 4 represents the mean of latest lipid parameters among diabetic vs. non-diabetic patients.

**Table 2:** Mean of latest lipid parameters. TC: total cholesterol: LDL: low-density lipoprotein; HDL: high-density lipoprotein; TG: triglycerides.

	Total		Low risk		High risk		p-value
	Mean	±SD	Mean	±SD	Mean	±SD	
TC (no = 360)	4.569	0.986	5.132	1.012	4.444	0.936	0.000
LDL (no = 362)	2.783	0.850	3.235	0.917	2.682	0.802	0.000
HDL (no = 360)	1.349	4.370	1.192	0.287	1.107	0.263	0.03
TG (no = 352)	1.622	0.907	1.599	0.676	1.592	0.751	0.947

**Table 3:** Lipid profile change over three readings. TC: total cholesterol: LDL: low-density lipoprotein; HDL: high-density lipoprotein; TG: triglycerides.

	Mean	Median	±SD	Minimum	Maximum
TC (no = 360)	-0.186	-0.1150	0.986	-4.77	3.22
LDL (no = 362)	-0.159	-0.1000	0.892	-4.32	3.27
HDL (no = 360)	0.024	0.0000	0.180	-1.00	1.34
TG (no = 352)	-0.033	0.0000	0.597	-4.00	1.90

**Table 4:** Mean of latest lipid parameters among diabetic vs. non-diabetic patients. TC: total cholesterol: LDL: low-density lipoprotein; HDL: high-density lipoprotein; TG: triglycerides.

	Diabetics			Nondiabetics		
	Mean	±SD	p-value	Mean	±SD	p-value
TC	4.391	0.948	0.000	4.942	0.962	0.000
LDL	2.641	0.813	0.000	3.081	0.854	0.000
HDL	1.098	0.266	0.012	1.175	0.271	0.011
TG	1.611	0.765	0.495	1.556	0.677	0.513

No statistically significant difference was found between males and females in the increment of change in all lipid profile parameters. Moreover, no statistically significant difference was found between patients with or without other comorbidities.

Regarding clinic visits, the magnitude of reduction favored patients who had ten or more visits to family medicine clinics, but it was not statistically significant. The same was seen in the number of referrals to health educator or dietician except for the TG level, which had a p-value of 0.002.

Patients who were shifted from one statin to another had more reduction in their TC, LDL, and TG than patients who remained on the same statin throughout the follow-up period, but that was not statistically significant (p-value > 0.05). Patients who were on statin plus fenofibrate when compared to patients who were on one statin only had a more significant reduction in their TC with a (-0.50 vs. -0.17) mean of change and a p-value of 0.137, LDL (-0.50 vs. -0.14) with p-value of 0.068 and TG (-0.38 vs. -0.01) with p-value of 0.008.

## Discussion

We set out this cross-sectional chart review study to assess the level of control of lipid profile among dyslipidemia patients at King Abdul-Aziz Medical City-National Guard (KAMC-NG) Riyadh, Saudi Arabia. Overall, there was a reduction of all lipid parameters over the study follow-up period. At the end of the study period, the mean LDL-C level was found to be 2.783 mmol/L for the total study sample, 3.235 mmol/L for the low-risk patients, and 2.682 mmol/L for the high-risk patients. This is considered uncontrolled since the targets for the low-risk and high-risk patients are <2.5 mmol/L and < 1.8 mmol/L, respectively, according to the latest guideline’s recommendations [7].

Published data by Al-Hassan et al. in 2018 found the mean LDL-C value in a group of the general Saudi population to be 3.11 ± 0.92 mmol/L [10]. Similarly, another local study for lipid profile in the general population, but for younger ages, with a mean age of 20.727, found the mean LDL-C level to be 2.57 ± 0.724 [17]. The different study populations of both studies compared to the present study should be considered in case of comparison.

Al‑Shehri studied the lipids profile of 295 angiographically documented coronary artery disease Saudi patients with a mean age of 55.1 ± 11 and found the mean total cholesterol to be 4.54 ±1.23 mmol/L and the mean LDL-C level to be 2.87 ± 1.04 mmol/L [17]. Those findings are comparable with the figures of high-risk patients in the present study.

In a Chinese study, with a large sample size of 163,641 participants of mixed population, high and low risk, the mean of TC and LDL-C was 4.7 and 2.88 mmol/L, respectively, and among individuals with high ASCVD risk, 74.5% had uncontrolled LDL-C levels [18]. When comparing initial lipid parameters with the latest, we can see the change in favor of control of dyslipidemia, although it was not good enough to bring the figures to optimal control.

Although dyslipidemia has been associated with obesity with increased susceptibility to coronary heart disease (CHD) two to three times more in obese adults [19,20], evidence suggests that dyslipidemia can be found in both obese and non-obese persons [21]. This has been noticed in our study since many participants are non-obese with either normal or overweight.

It was observed in our study that the majority of the patients had diabetes mellitus. Evidence confirmed that diabetes is associated with a high incidence of dyslipidemia [22]. Furthermore, a study suggested that HbA1c can be used as a predictor of dyslipidemia and thus early diagnosis in patients with type 2 diabetes mellitus (T2DM) [23].

On average, the patients were obese with a mean BMI of 32.3 kg/m^2^ ± 5.87 kg/m^2^. The prevalence of obesity among current study participants, with a mean BMI of 32.3 kg/m^2^, goes with the latest estimated prevalence of obesity among the Saudi population as reported by the World Health Organization (WHO) to be 35.4% [24].

Females in our study constitute the simple majority of patients with dyslipidemia. That can be understood because obesity is more prevalent among females in Saudi Arabia. This needs to be investigated further since previous studies showed that the prevalence of dyslipidemia is more in males than females [25,26].

A healthy lifestyle was encouraged and emphasized as the first line of treatment for dyslipidemia in recent guidelines [7]. Boyer et al. reported that a one-year lifestyle intervention program aiming to increase physical activity levels and improve diet quality positively impacted several cardiometabolic risk factors in men with dyslipidemia [27], even a moderate lifestyle modification intervention is effective [28].

Evidence showed that a patient's motivation could positively impact the reduction in lipid parameters, adherence to a healthy lifestyle, and medication compliance. Frequent visits to primary care physicians can achieve this motivation [29]. This comes in agreement with our result as patients who had more than ten visits to FM clinic and visited dietician and health educator had a significant reduction in their lipid parameters.

Although dyslipidemia is more common in males than females, as reported by several international studies[25,26], males in our study have significantly decreased their LDL over the study period more than females. Per our findings, a previous study that assessed age- and gender-related differences in LDL-C management in outpatients with T2DM came out with the findings that females were always monitored less frequently than males, irrespective of age [30]. Similarly, Zhang et al., in their study, found that the percentages of patients who achieved the LDL-C goal were higher among males [31].

In the current study, patients with diabetes showed better control of LDL-C than others. This may be attributed to frequent follow-ups and a more multidisciplinary approach for patients with diabetes. A study done in China showed a resemblance in the level of control among patients with diabetes [32].

To date, statins remain the first-choice therapy for the prevention and treatment of dyslipidemia since they have been shown to reduce the annual major vascular events risk by 21% [33,34]. Study by Shuhaili MF et al. demonstrated that statins reduced LDL-C, TC, and TG, and the reduction was more with atorvastatin compared to simvastatin, pravastatin, lovastatin [35]. Such findings are similar to ours, where patients in the current study who achieved a reduction in lipid parameters, almost all of them were using statins, and atorvastatin was the most commonly used one. It is recognized that atorvastatin is the most extensively utilized statin as it is a much safer and endured lipid-lowering therapy compared to other statins and with fewer side effects [36].

A review of guidelines and clinical trials assessing non-statin agents explains the evidence and expert opinion recommending the use of combination lipid-lowering treatments [37]. It was suggested that therapy with a fibrate-statin combination produces clinical outcomes that are higher to treatment with fibrate alone but raises the chance of side effects (predominantly renal). Therefore the necessity for monitoring during treatment. [38]

The results of previous studies confirmed the efficacy of fenofibrate and Statin combination. It was found that combination therapy in patients with dyslipidemia and type 2 diabetes was tolerated and succeeded in improving the lipid parameters than either monotherapy [39]. Similar findings were obtained from our study since patients who were on statin plus fenofibrate had a greater reduction in their TC, LDL, and TG. In another study, ezetimibe was superior to other non-statin therapies when added to a statin in terms of lowering LDL safely and showing further clinical benefit in patients at high risk of ASCVD [40].

One of the study limitations is that stratifying participants in this study for ASCVD risk was based only on the presence of DM and HTN, not according to risk calculators due to lack of information about other risk factors, In addition, compliance to medication was not assessed.

## Conclusions

Most of the patients visiting the Ministry of National Guard - Health Affairs (MNG-HA), Riyadh, Saudi Arabia, showed a continuous reduction in their lipid profile over the study period; however, high-risk patients showed better control when classified into low risk and high risk. In addition, many factors may have contributed to the level of reduction, like the number of clinics, dieticians, health educator visits, and the type of medication used. There is a necessity for carrying special measures to deal with dyslipidemia as a health priority. More intensive treatment of dyslipidemia in female and high-risk patients with comorbidities should be applied and continuous lifestyle modification, with frequent follow-ups and lipid profiles monitoring. As it is a multifactorial disease, dyslipidemia needs a more comprehensive team-based collaborative approach for better control.
